# Resilience: Biological Basis and Clinical Significance — A Perspective Report from the International Conference on Frailty and Sarcopenia Research (ICFSR) Task Force

**DOI:** 10.14283/jfa.2022.62

**Published:** 2022-10-27

**Authors:** Matteo Cesari, D. Azzolino, N. K. LeBrasseur, H. Whitson, D. Rooks, S. Sourdet, D. Angioni, R. A. Fielding, B. Vellas, Y. Rolland, Sandrine Andrieu, Mylène Aubertin Leheudre, Nuria Barcons, Ann Beliën, Philipe de Souto Barreto, Carla Delannoy, Groarke John, Luis Miguel Gutierrez Robledo, Darren Hwee, Jean Mariani, Merchant Reshma, John Morley, Suzette Pereira, Quann Erin, Rossulek Michelle, Ricardo Rueda, Lisa Tarasenko, Cendrine Tourette, Rob Van Maanen, Debra L. Waters

**Affiliations:** 1 Geriatric Unit, IRCCS Istituti Clinici Scientifici Maugeri, University of Milan, Via Camaldoli 64, 20138 Milano, Italy; 2 Robert and Arlene Kodod Center on Aging, Department of Physical Medicine and Rehabilitation, Mayo Clinic, Rochester, USA; 3 Duke University School of Medicine & Durham VA Medical Center, Durham, USA; 4 Translational Medicine, Novartis Institutes for Biomedical Research Inc., Cambridge, USA; 5 Gérontopôle de Toulouse, Centre Hospitalier-Universitaire de Toulouse, Inserm 1295, Université de Toulouse, Toulouse, France; 6 Nutrition, Exercise Physiology, and Sarcopenia Laboratory, Jean Mayer USDA, Human Nutrition Research Center on Aging at Tufts University, Boston, MA USA; 7 Toulouse, France; 8 Montreal, Canada; 9 Barcelona, Spain; 10 Heusden-Zolder, Belgium; 11 Vevey, Switzerland; 12 New York, USA; 13 Mexico City, Mexico; 14 South San Francisco, USA; 15 Paris, France; 16 Singapore, Singapore; 17 St. Louis, USA; 18 Columbus, USA; 19 Dunedin, New Zealand; 20 Albuquerque, USA

**Keywords:** Aging, biomarkers, geroscience, older adults, translational research

## Abstract

The Resilience is a construct receiving growing attention from the scientific community in geriatrics and gerontology. Older adults show extremely heterogeneous (and often unpredictable) responses to stressors. Such heterogeneity can (at least partly) be explained by differences in resilience (i.e., the capacity of the organism to cope with stressors). The International Conference on Frailty and Sarcopenia Research (ICFSR) Task Force met in Boston (MA,USA) on April 20, 2022 to discuss the biological and clinical significance of resilience in older adults. The identification of persons with low resilience and the prompt intervention in this at-risk population may be critical to develop and implement preventive strategies against adverse events. Unfortunately, to date, it is still challenging to capture resilience, especially due to its dynamic nature encompassing biological, clinical, subjective, and socioeconomic factors. Opportunities to dynamically measure resilience were discussed during the ICFSR Task Force meeting, emphasizing potential biomarkers and areas of intervention. This article reports the results of the meeting and may serve to support future actions in the field.

## Introduction

The Resilience is “the ability to recover or optimize function in the face of age-related losses or disease” (1). Indeed, it determines the dynamic propensity of the organism to lose function and subsequently recover after the disruption of homeostasis due to one or more external factors. The study of physical resilience has been indicated as a priority by the US National Institute on Aging (2). In recent years, the construct of resilience has received growing attention, especially during the COVID-19 pandemic (3), which is a global stressor that has been critically implicated in accelerating the aging process (4).

Resilience is multidimensional in nature. In other words, the measurement of resilience may require the longitudinal assessment of multiple factors and cannot be captured by a single generalized test (5, 6). Different measures of resilience have been proposed (4, 6, 7), which tend to distinguish physical and psychological resilience.

The International Conference on Frailty and Sarcopenia Research (ICFSR) Task Force met in Boston (MA, USA) on April 20, 2022 to discuss the clinical and biological significance of resilience. The discussion was specifically focused on how to measure resilience in clinical and research settings. Potential strategies to advance the field were also debated. Preliminary results of ongoing research activities were presented. The present article summarizes the contents of the meeting to further promote the discussion and interactions on the topic.

## Resilience in frail older persons: clinical and biological aspects

The person’s resilience depends on his/her physiological and clinical capacities/reserves, allowing him/her to adapt to stressors (8). Environmental factors (including social support) may also be considered a contributor to an individual’s resilience as representing significant determinants of the ability to cope with stressors.

The static assessment of the person’s health is a good, but limited, predictor of his/her capacity to recover after a disruptive event. Whereas capturing the dynamic modifications through longitudinal monitoring allows one to measure the robustness of the biological and homeostatic states. In fact, single health assessments (e.g., static measures of disease burden or frailty) are associated with incident adverse events, but the predictive capacity is improved when multiple assessments are combined in the definition of a health trajectory (9–11).

Unlike young individuals, who have a high level of recovery following stressful events, older persons have a lower and heterogeneous capacity to “bounce back” to pre-stress functional status. In other words, the aging process determines a change of the so-called tipping point (i.e., the threshold defining the homeostatic equilibrium of the organism), consequently varying the capacity to cope with stressors. The identification and assessment of the tipping point may provide a dynamic measure of resilience and inform the design of preventive/therapeutical interventions (9).

Recently, Guion et al. (12) described four distinct trajectories of recovery in nursing home (NH) residents admitted to the emergency department. In particular, the health trajectory was drawn observing the changes of physical performance from the pre-event status to the return to the NH. Those NH residents with the highest level of physical reserves at the baseline showed the best capacity for recovery. On the other hand, those with the worst status at the baseline presented minimal capacity for recovery. However, it is noteworthy that, whereas models like this tend to be quite reliable when observing a population/group, their robustness is substantially lower when applied to the individual level. In fact, the inter-individual variability can significantly affect and deviate the trajectories from expectations. Indeed, there is a need for more than just the assessment of the initial reserves when exploring the inner capacities and reserves of the person.

Chronological age is an essential factor associated with resilience. Applying multi-state modeling approach to estimate 1-year transition probabilities of the New Mexico Aging Process Study data (13), the decline of walking speed and cognitive function among healthy persons was prospectively described over a follow-up of 9 years. The rate of decline experienced by the youngest part of the population (i.e., people aged 60 to 78 years) was relatively small; at the same time, a high capacity for recovery was observed. On the other hand, in persons older than 78 years, a two-fold risk of decline and a halved chance of recovery was estimated after an adverse event.

A small study (14) investigated the effects of two weeks of immobilization followed by four weeks of retraining on muscle function and fiber morphology in a sample of healthy men with similar levels of physical activity, according to age (n=9 older vs. 11 younger individuals). It was reported that older participants were more susceptible to the adverse effects of short-term muscle disuse, in terms of muscle fiber size and rapid force capacity, compared to the younger counterparts. Furthermore, older persons required a longer time for muscle function recovery.

Age-related changes in resilience are likely mediated by biological processes. As suggested by López-Otín and colleagues (15), different strategies are used by organisms to attain biological stability. These strategies are based on homeostatic resilience (i.e., genetic, neural, metabolic, immunological, microbiome-based mechanisms), hormetic regulation (i.e., mitohormesis, healthspan, lifespan), and repairing and regeneration capacities. Indeed, it is crucial to investigate the so-called “hallmarks of aging”, especially given the hypothesis that loss of resilience anticipates the clinical onset of frailty. It would thus be possible that biological tests might predict future health events and guide geroprotective interventions by targeting the hallmarks of aging and modulating the organism’s resilience.

During the Task Force meeting, preliminary results from secondary analyses conducted in the Multidomain Alzheimer Preventive Trial (MAPT) (16) database were presented. The analyses were aimed at investigating the longitudinal relationship of mitochondrial function, regeneration, and inflammation with frailty. Physical frailty was measured according to the phenotypic criteria proposed by Fried and colleagues (17) at the study baseline and every year for two years. Different biomarkers of aging, inflammation, and mitochondrial function (e.g., C-Reactive Protein [CRP], Interleukin-6 [IL-6], Tumor Necrosis Factor Receptor-1 [TNFR-1], Monocyte Chemoattractant Protein-1 [MCP-1], Growth Differentiation Factor-15 [GDF-15], Periostin) were measured. Results showed that those participants who became frail after 12 months had higher levels of TNFR-1 and GDF-15 at the baseline. Of these, participants who reversed to robust after 12 additional months had lower GDF-15 concentrations, a promising biomarker of cellular aging and systemic inflammation (18–20), compared to those who remained frail. The analyses presented limitations (e.g., a small number of frail participants). However, the results were still suggestive of the role that biomarkers of aging may play in the definition of the recovery process.

Kirkland et al. (21) proposed several stressors for potentially assessing the physiological resilience in animal models (i.e., starvation, water deprivation, anesthesia, chemotherapy, trauma, temperature stress, cortisol, circadian rhythm, infection, barbiturates). In daily practice, many other situations can be envisioned as stressful events (e.g., sepsis, infections, vaccinations, immobilization and bed rest, chemotherapy, altered diet, dehydration, surgical stress (22)) to use for studying and measuring resilience. In particular, the recovery from surgical stress may represent a very interesting benchmark to advance in the field, mainly because it is easier to fix in the temporal sequence of the health trajectory. Nevertheless, the main problem in clinical practice is that resilience is diagnosed/seen a posteriori. Consequently, it is complicated to predict whether the older person will recover or not.

The targeting of biological mechanisms with geroscience-based interventions, including pharmacological (i.e., metformin, angiotensin receptor blockers, dasantinib, quercetin), hormonal (i.e., oxytocin), physical (i.e., brain and muscle stimulation) and nutraceutical (i.e., Vitamin D, resveratrol, Omega-3) strategies, represents a promising frontier in the attempt to preserve and improve mobility function in older people (23, 24). In the context of infectious diseases (e.g., COVID-19, influenza, pneumonia), there might also be opportunities to improve the immunological status while acting on the background biology of aging through pharmacological interventions (e.g., low-dose mammalian Target of Rapamycin [mTOR] inhibitors) (25). Chemotherapies may also cause accelerated aging-like states. In this situation, it could be investigated the potential of senotherapeutic drugs that selectively induce apoptosis of senescent cells (i.e., senolytics) or suppress their secretory phenotype (i.e., senomorphics) (26).

Strategies integrating resilience in the daily routine of geriatric medicine are also amenable. The Integrated Care for Older People (ICOPE) initiative proposed by the World Health Organization (WHO) might represent an opportunity in this direction as designed to reshape the care models toward a person-centered approach (27, 28). For example, the Gérontopôle of Toulouse (France) has recently implemented clinical activities based on the ICOPE model (29). Persons included in the program may be asked to be part of research programs devoted to 1) the study of health trajectories and dynamics, and 2) the exploration of possible geroprotectors. In this context, digital medicine and the use of remote monitoring of health parameters open up considerable prospects. The longitudinal analysis of big data in the general population could make it possible to identify in the future subjects with low resilience and implement targeted preventive interventions.

## Perspectives on Resilience

Several mechanisms are involved in the aging process, representing the biological substratum of many age-related conditions (e.g., frailty, sarcopenia, cancer, neurodegenerative conditions, immunological disorders, cardiovascular diseases). It has been hypothesized that interventions targeting such background may prevent the onset of typical conditions of old age and represent a potential opportunity to extend human healthspan through the compression of morbidity.

In this context, resilience is an interesting construct to explore the biology of aging because it captures the dynamic system of the organism over time (potentially even before conception; i.e., in utero) (30–32). In other words, an impaired resilience might be considered a marker of accelerated aging, becoming a clinically relevant expression to capture before an overt manifestation (30, 33). At the same time, a compromised resilience may enhance the biological aging, feeding the generation of a vicious cycle.

The measurement of resilience may support the study of the individual’s health trajectory and provide a means for the early identification of those at risk of decline (34). There might indeed be a window for remarkable opportunities to preventively improve the health status by acting on the biology of aging. For example, rapamycin has been shown to extend life span and ameliorate resilience towards age-related conditions (including immune dysfunction) via the inhibition of the mTOR pathway (35–37). During the COVID-19 pandemic, many have hypothesized a role for interventions able to boost or improve resilience in frail older persons at risk of suffering the most severe consequences of the viral infection. Senotherapeutic compounds have also been proposed to support the individual exposed to the pathogenic stress of the severe acute respiratory syndrome due to SARS-CoV-2 infection (38). A potential role of them has also been hypothesized in the long-COVID syndrome (39).

A paradigm for translation is represented by the design of clinical trials to test geroprotective agents. Most funded clinical trials are designed to provide straightforward answers following the so-called “standalone-disease” approach, which investigates and treats a well-defined condition in a sort of isolation from the complexity of the aging process (40). The Targeting Aging with Metformin (TAME) study, a proof-of-concept clinical trial designed to look at the effect of metformin on the incidence of age-related diseases, is an example of how to describe the effects of an intervention on health trajectories before the occurrence of overt diseases (41, 42).

The idea of modeling resilience in animal models to translate results into humans is fascinating. Indeed, resilience can be explored in animals in a relatively simple way and adopting a holistic approach. Interestingly, mice of similar age have shown a broad spectrum of responses after stressors are applied, suggesting the possibility of using them in the study of resilience (21).

The implementation of resilience in the clinical setting requires the development of feasible and meaningful assessment instruments. Preliminary results of a study conducted in genetically heterogeneous female and male mice aimed at exploring if midlife resilience could be predictive of lifespan were anticipated during the Task Force meeting. It was shown that resilient mice presented a longer life expectancy, especially in females. It was also demonstrated that resilience in midlife was associated with better physical, metabolic, and cardiac function at a more advanced age, especially in males. The findings suggest that interventions applied earlier in life or before stressful events (e.g., prehabilitation) might be beneficial.

**Figure 1:**
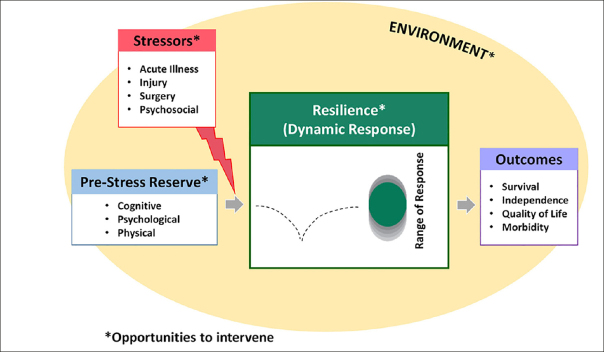
Duke University Pepper Center conceptual model of resilience Reproduced from Whitson et al. (J Am Geriatr Soc 2021;69:3232-41) under a Creative Commons Attribution Non-Commercial (CC BY-NC) license.

In the Duke University Pepper Center conceptual model of resilience (Figure 1), the pre-stress reserve comprises multiple domains, including psychological, physiological, and cognitive abilities that each person accesses to adapt in the face of a health stressor. At the same time, two different conceptual approaches have been proposed to quantify resilience after a stressor (43). The first one (i.e., the recovery phenotype) is based on observing the individual recovery patterns across health measures over time. This approach is a highly descriptive, multiparametric model that can simultaneously consider multiple outcomes (i.e., latent class trajectory analysis, factor analysis, principal components analysis). Age, comorbidities, and pre-stressor function highly drive the recovery phenotype. The phenotypic way to quantify resilience is useful for prognostic models in clinical practice or classifying outcomes in intervention research. For example, in a recent study, the recovery phenotype approach has been used as a model to study resilience in older adults following a hip fracture (44). In particular, the recovery trajectories for multiple selected outcomes (i.e., daily steps count, time to complete single chair stands, grip strength, gait speed) were described in three different resilience groups (i.e., low, medium, and high resilience). The authors found that the pre-stressor functional status was the strongest predictor of the subsequent recovery.

**Figure 2:**
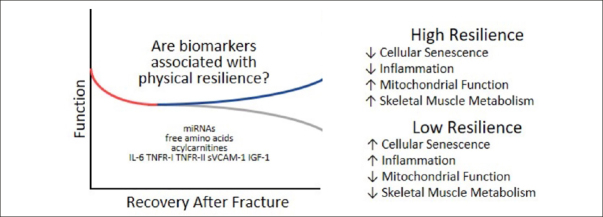
Different recovery patterns after a fracture event miRNAs: microRNAs; TNFR-I: tumor necrosis factor-α receptor I, TNFR-II: tumor necrosis factor-α receptor II, sVCAM-1: soluble vascular adhesion molecule-1; IL-6: interleukin-6; IGF-1: Insulin Growth factor-1

Another way to quantify the clinical trajectory of recovery is represented by the so-called “expected recovery differential approach”, which is aimed at quantifying how observed outcomes differ from expected outcomes. This approach, based on predictive models performed in large cohorts, accounts for baseline status, stressor-related factors, and environment. It might be particularly appropriate to identify the biological mechanisms underlying resilience (43). The expected recovery differential was recently applied by Parker et al. (45) to identify biomarkers of resilience after hip fracture in older adults. Different biomarkers of aging representing the inflammatory and immunological pathways (i.e., TNFR-1, TNFR-2, soluble vascular adhesion molecule-1 [sVCAM-1], and IL-6), the metabolic and mitochondrial function (i.e., non-esterified fatty acids, lactate, ketones, acylcarnitines, free amino acids, and insulin-like growth factor 1), and the gene expression (i.e., circulating microRNAs [miRNAs]) were evaluated. It was found that the full panel of biomarkers explained 38% of the resilience variance, defined as expected recovery differential. After performing a principal component analysis on the 64 metabolites, four factors were identified. In particular, the most parsimonious set predicting the expected recovery differential (generated by a Least Absolute Shrinkage and Selection Operator [LASSO] regression) explained 27% of resilience variance after hip fracture. These results are in accordance with the hypothesis that those people showing a better recovery after a hip fracture present a lower degree of cellular senescence, inflammation, mitochondrial dysfunction, and muscle impairment (Figure 2) in the days close to the event.

Finally, during the Task Force meeting, the protocol of the PRIME-KNEE study (46) was presented. PRIME-KNEE is a prospective cohort study enrolling 250 patients aged 60 years and older scheduled for elective knee replacement surgery. The primary objective of the PRIME-KNEE study is to validate provocative tests and biomarkers predicting recovery in the perioperative period. For this purpose, participants are assessed for cognitive, physical, and psycho-social reserves during the baseline visit. Moreover, they also undergo a series of provocative tests (i.e., dual-task effect on gait speed, cerebrovascular reactivity assessed by functional near-infrared spectroscopy, peripheral blood mononuclear cells reactivity tests) and blood drawn to assess biomarkers of interest. A follow-up, including in-person visit after six months from surgery and several other measurements (i.e., pain intensity and inference, cognitive change index, 7-day step counts, rehabilitative procedures), is also planned.

## Conclusions

Resilience represents a promising area of investigation for research on aging. The biological and clinical understanding of resilience will allow the development of targeted interventions for its improvement. In particular, it will be necessary to 1) define the best way to identify individuals with low resilience and 2) describe the correct methodology for promptly intervening before the onset of adverse events.

The ICFSR Task Force concluded that, given the involvement of multiple organs and systems, measures of resilience should be multidimensional and consider a broad spectrum of outcomes. At present, holistic interventions improving physical, psychological, and cognitive function seem particularly promising to boost resilience. The identification of biomarkers of resilience represents a necessary strategy to improve the understanding of the heterogeneous health trajectories following stressful events.
